# Temperate mountain grasslands: a climate-herbivore hypothesis for origins and persistence

**DOI:** 10.1111/brv.12063

**Published:** 2013-10-04

**Authors:** Peter D Weigl, Travis W Knowles

**Affiliations:** 1 Department of Biology, Wake Forest University1834 Wake Forest Road, Winston-Salem, NC, 27106, U.S.A.; 2 Department of Biology, Francis Marion UniversityPO Box 100547, Florence, SC, 29502-0547, U.S.A.

**Keywords:** disturbance regime, East Carpathians, grass bald, keystone herbivores, landscape conservation, Oregon Coast Range, palaeoecology, Pleistocene, polonina, Southern Appalachians

## Abstract

Temperate montane grasslands and their unique biotas are declining worldwide as they are increasingly being invaded by forests. The origin and persistence of these landscapes have been the focus of such controversy that in many areas their conservation is in doubt. In the USA some biologists have largely dismissed the grass balds of the Southern Appalachians as human artifacts or anomalous and transitory elements of regional geography, worthy of only limited preservation efforts. On the basis of information from biogeography, community ecology, regional history and palaeontology and from consideration of two other montane grassland ecosystems—East Carpathian poloninas and Oregon Coast Range grass balds—we hypothesize that these landscapes are more widespread than was formerly recognized; they are, in many cases, natural and ancient and largely owe their origin and persistence to past climatic extremes and the activities of large mammalian herbivores.

## CONTENTS

- Introduction 467
- Southern Appalachian balds 467
- Hypothesis 468
  1. The case for the climate–herbivore hypothesis 468
    - Historical eastern USA grasslands 468
    - Palaeobotanical and palynological evidence 469
    - Questionable Native American influence 469
    - European settlement history 469
    - Rare, relictual and endemic plant community 469
    - Extant grass bald fauna 469
    - Current dependence on mowing and grazing management 469
    - Pleistocene megaherbivores and the ecological role of extant keystone herbivores 470
  2. The loss of the megafauna 471
  3. Criticisms of the grazing hypothesis 471
- Additional candidate ecosystems 471
  1. East Carpathian poloninas 471
  2. Oregon Coast Range balds 473
- Current status and further tests of the climate–herbivore hypothesis 473
- Conclusions 474
- Acknowledgements 474
- References 474

## I. INTRODUCTION

Temperate montane grasslands and their associated rare, endemic and relict biota are declining worldwide (Zald, 2009). Mountain meadows, often known as grass balds, with long histories of open expanses are now being invaded and replaced by forests. These communities have been the focus of much research and debate regarding their origins, longevity, and landscape dynamics (Miller & Halpern, 1998; Weigl & Knowles, 1999; Wiser & White, 1999; Zald, 2009). Herein we compare and synthesize information from three such mountain ecosystems—Southern Appalachian grass balds, East Carpathian poloninas, and Oregon Coast Range grass balds—and propose a new unified hypothesis for their origins and persistence involving climate and herbivory. Such an hypothesis emphasizes an historical context for these landscapes and their biotas, incorporates insights from other grassland systems and provides an argument for future research and conservation. Since questions about the origin and persistence of Appalachian balds have an especially long history and are associated with an extensive literature, we place greater emphasis initially on this region and then focus on the similar ecological patterns in the other two grassland ecosystems.

The three candidate ecosystems discussed herein share several common features: (*i*) each is a montane, gramminoid community located below present-day climatic treeline. (*ii*) Each hosts a diverse herbaceous community marked by light-dependent (i.e. shade-intolerant) species, suggesting a long, shade-free history. (*iii*) All are located at northern latitudes, but south of maximal Pleistocene ice coverage, which likely repeatedly limited the elevational range of forest vegetation. (*iv*) Each is within the well-established range of a diverse Pleistocene mammalian megafauna, which grazed and browsed these regions, until reduced in diversity and body size at the time of the Holocene extinctions. (*v*) All experienced modern grazing by domestic animals, with loss of grassland area to woody invasion after grazing ceased.

We propose then that two major factors—the suppression of forest by glacial climate, followed by the ecosystem engineering of megaherbivores and their mid-sized successors—best explain these ecosystems as modern remnants of formerly more widespread open habitats. Such an idea is entirely consistent with the now well-established role of mammalian herbivory as a major top-down influence on vegetation structure and geography in many other ecosystems worldwide (Bond, 2010; Marquis, 2010).

## II. SOUTHERN APPALACHIAN BALDS

The grass balds of the Southern Appalachians are among the more spectacular natural features of the eastern United States, occupying isolated peaks and ridges from the Great Smoky Mountains, through North Carolina, Tennessee and Virginia to southern West Virginia (latitude 35°30′ to 39°30′: elevation mostly 1220–1830 m) (Mark, 1958; Gersmehl, 1970; Rentch & Fortney, 1997; White, 2006). Grass balds are characteristically areas of naturally occurring, treeless vegetation dominated by grasses, sedges and forbs and located on well-drained sites below climatic treeline in predominantly forested regions (Mark, 1958). Over decades, a variety of environmental and anthropogenic factors have been proposed for bald origin and maintenance, including contemporary climate, topography, soils, severe weather, fire, clearing by Native Americans or early European settlers, and post-glacial warming; none have borne up to scientific scrutiny (White & Sutter, 1999).

In the Appalachians, these communities are often surrounded by forests of red spruce (*Picea rubens*), Fraser fir (*Abies fraseri*), beech (*Fagus grandifolia*), and yellow birch (*Betula alleghaniensis*), and in some cases, stands of red oak (*Quercus rubra*), chestnut oak (*Q. montana*), buckeye (*Aesculus flava*) and a few smaller tree species. Along with the rocky outcrops imbedded within them, the balds are famous for their biotic diversity, scenic beauty and their frequent mention in the accounts of early explorers (Laughlin, 1999). At one time balds were both more numerous—up to 100 were recognized by Gersmehl (1970)—and more extensive, covering whole ridgelines according to the descriptions of early European explorers (Laughlin, 1999). In the mid-19th and much of the 20th century they were used as grazing lands by some of the descendents of the original settlers. Now the balds are declining. After years of little apparent change, they are being invaded by weedy intruders and neighbouring forests, and their persistence in many areas is in doubt.

During the past 70 years some biologists and government agencies have tended to dismiss the balds as human artifacts—the result of relatively recent Native American activities, perhaps—or, more likely, the agricultural practices of European settlers (Gersmehl, 1970, 1973). Implicit in this view is the assumption that the eastern USA was once largely continuous forest with few grasslands until the advent of agriculture and that grass balds should be considered anomalous, isolated and transitory artifacts of Appalachian landscape history. Balds, according to this artifact hypothesis, are merely disjunct points of interest, not natural landscapes, and are worthy of only limited conservation efforts—to preserve rare plant species, some special vistas and a few sites of historical interest (Gersmehl, 1970; Lindsay & Bratton, 1979; Sutter & White, 1994).

In contrast to this ‘artifact’ interpretation, we propose a climate–herbivore hypothesis to explain bald origins and persistence. We maintain that many of these communities, while often modified by human activity over the last 150 years, are very likely natural and the ancient product of Pleistocene climatic changes initially, and subsequently the feeding activities of a diverse group of large grazers and browsers, possibly along with occasional fires (Weigl & Knowles, 1995, 1999). The resulting disjunct array of high-elevation grasslands may accordingly explain the evolution, persistence and dispersal of a distinctive biota intolerant of closed forest habitats. Indeed, the recent absence of almost all these keystone herbivores may be a major factor in bald disappearance. This ‘climate–herbivore hypothesis’ emphasizes the importance of dynamic ecological and evolutionary processes over long periods of time and lends support to policies that would preserve whole bald landscapes and their dependent biota.

For many years the controversy between an ‘artifact’ hypothesis and a ‘climate–herbivore’ hypothesis as explanations of grass bald origins and persistence inhibited substantive conservation or restoration efforts. In the meantime the balds continued to disappear. Recently, in a response to a new awareness of the potential loss of a landscape and biota, biologists working in the Great Smoky Mountains, at Roan Mountain and a few other sites have started to restore bald areas (White & Sutter, 1999). However, it will take a major change in attitude towards, and understanding of, bald ecosystems to provide the impetus and rationale for bald protection.

## III. HYPOTHESIS

On the basis of information from many sources (enumerated below), we advance the idea that grassy areas have persisted on the upper elevations of some of the Appalachians from at least the late Pleistocene. These open landscapes were initially created by the severe climatic conditions associated with glacial advances, which displaced woody vegetation from peaks and ridges (Delcourt & Delcourt, 1988). The resulting open gramminoid communities or forest-gramminoid mosaics were maintained by a diverse group of now mostly extinct browsers and grazers until perhaps 11500 calendar years before present (y BP) (Russell *et al.*, 2009), followed by a much reduced herbivore fauna until European settlement and then by pastoral activities during much of the last two centuries. Thus, the distinctive animal and plant communities of today's grass balds are likely mere remnants of a once more extensive landscape, which resulted from the long-term disturbance regime of severe climate, herbivory and perhaps occasional fire. These communities disappear in the absence of natural disturbance, through succession to woody vegetation. A similar ecological sequence may well help explain the existence of grasslands on other temperate mountains such as the poloninas of the Carpathian Mountains and the grass balds of the Oregon Coast Range.

This hypothesis comes with a few caveats. We in no way wish to insist that all current mountain grassy areas had the same origin and history. Based on historical records, some sites called ‘balds’ are clearly products of human activity in the late 19th and 20th centuries (Lindsay, 1976, 1978; Lindsay & Bratton, 1979), and almost all balds have been modified by local people in the last 200 years. We also emphasize the role of herbivores because of limited evidence for extensive and frequent fires at these elevations in the past (Barden, 1974; Lindsay, 1976; Delcourt & Delcourt, 1997; Knoepp, Tieszen & Fredlund, 1998; White & Sutter, 1999; Copenheaver *et al.*, 2004; Crawford & Kennedy, 2009; Fesenmyer & Christensen, 2010) and the existing problems today in the use of fire as a management tool due to cool, moist conditions (Murdock, 1986). That fire, along with grazing, might have been a factor during periods of warm, dry conditions is entirely possible and may be established by future research. We are also aware of ‘bottom up’ and ‘edaphic patch’ (i.e. resource limitation) explanations for some grasslands and plant distributions in the southeastern USA (Baskin & Baskin, 1988; Paine, 2000; Owen, 2002); however, we suggest that factors such as site, soil and substrate diversity are of lesser importance than light dependence for much of the bald biota (see Baskin & Baskin, 1988; White & Sutter, 1999). In addition, while we recognize the extensive literature on the mechanisms of tree invasion into grass balds, involving processes of seed dispersal, facilitation, competition and various physical stresses (Magee & Antos, 1992; Johnson & Smith, 2005; Dovčiak *et al.*, 2008; Halpern *et al.*, 2010), the emphasis of these studies has been on mechanisms and rates of woody species colonization of grasslands. By contrast, our major concern is with those disturbance factors that might halt or reverse such encroachment. Finally, although many plant ecologists consider the species-rich rocky outcrops of these grasslands as separate communities unrelated to the balds, we have observed that many outcrops indeed lie within balds, support light-dependent plants, and are also degraded or destroyed by forest encroachment and shading. Thus, they are included in our discussion here.

### (1) The case for the climate–herbivore hypothesis

Many of the arguments for this interpretation of grass bald history and ecology have been presented in earlier publications (Weigl & Knowles, 1995, 1999) and thus will be summarized here.

#### (a) Historical eastern USA grasslands

The idea that the natural vegetation of the eastern USA was exclusively, or even largely, forest during the Pleistocene and Holocene is now untenable as coastal plain savannas (DeSelm & Murdock, 1993; Juras, 1997), Piedmont prairies (Taecker, 2007), the limestone glades and barrens in Tennessee, Kentucky and adjacent states (Baskin, Baskin & Chester, 1994), canebrakes (Platt & Brantley, 1997) and prairie peninsulas (Gordon, 1969; Taft, 1997) become better documented (Noss, 2013) and as the descriptions of early explorers (e.g. Lawson, 1984; Bartram, 1998) are rediscovered. Grasslands and unforested areas were thus common components of past eastern USA landscapes (Mac *et al.*, 1998; Carroll *et al.*, 2002; Owen, 2002; Copenheaver *et al.*, 2004; Russell *et al.*, 2009; Noss, 2013).

#### (b) Palaeobotanical and palynological evidence

Vegetation reconstructions by palaeobotanists suggest that periods of glacial advance were associated with climates so severe on mountain peaks that forest vegetation was driven downslope and replaced by grasslands or tundra (Delcourt & Delcourt, 1984, 1988)—terrain essential to light-requiring plants and beneficial to an array of cold-adapted herbivores. There is also evidence from palynological data that some high elevation sites did not support forests 3000 years ago (Shafer, 1986).

#### (c) Questionable Native American influence

There is virtually no evidence of substantial Native American modification of these peaks by clearing or fire, as was proposed by Wells (1936, 1938, 1956). Most recent work emphasizes human impacts at lower elevations rather than on the peaks and ridges (Delcourt & Delcourt, 1997; White & Sutter, 1999). Grass balds were undoubtedly used as hunting grounds and campsites, and may have at times been dry enough to burn, but were not suited for crops or permanent settlements (Smathers, 1981; Laughlin, 1999).

#### (d) European settlement history

The common assertion that early settlers created the balds to graze their livestock is not supported by historical evidence. Many of the balds are known to pre-date European settlement. In fact, bald descriptions go back to at least the 17th century (Weigl & Knowles, 1999). Before the 19th century the southern Appalachians were still largely dominated by Native American peoples, not by Europeans. Most settlers passed through or around the higher mountains to the broad valleys of southwestern Virginia and eastern Tennessee. Settler populations in the mountain areas were generally small. For example, the whole Toe River valley in North Carolina—an area near a number of the balds—had only 80 settler families by 1790. Most people lived in the lowlands as farmers, not pastoralists, and relied on hogs rather than grazers as meat animals because of the availability of abundant acorn and chestnut crops (Cooper, 1964). Grazing on the uplands did not really assume importance until the mid 19th century (Gersmehl, 1970; Smathers, 1981). Since it is unlikely that early settlers would clear vast areas on mountaintops far from home or drive their herds into the middle of dense forests, one might assume that the fact that these grasslands already existed was a major consideration in their eventual exploitation.

#### (e) Rare, relictual and endemic plant community

The balds support many rare, endemic, relict and disjunct plant species (Table 1) whose survival in viable populations requires the open terrain of the balds and their associated rocky areas (Baskin & Baskin, 1988; Schafale & Weakley, 1990; White & Sutter, 1999). These plants do not flourish under a closed canopy. Indeed, they disappear when the balds grow over. Such a group of light-dependent species must have had suitable conditions over a wide enough area and for sufficient time to speciate, to avoid the extinction effects of small population size, and to disperse along some of the ridges (Godt, Johnson & Hamrick, 1996). Interestingly, one of the dominant bald grasses, *Danthonia compressa*, occurs in a form that is considered the result of long-term grazing pressure (Clay, 1983).

**Table 1:** Representative rare, relictual and endemic herbaceous flora of the Southern Appalachian grass balds, USA *NatureServe Explorer* (Natureserve 2013)

Scientific name	Conservation status
Agrostis mertensii*	G5
Calamagrostis canadensis*	G5T5
Carex siccata*	G5
Carex cristatella*	G5
*Carex misera*	G3
*Carex oligosperma*	G5
*Carex ruthii*	G3
Delphinium exaltatum*	G3
*Deschampsia flexuosa*	G5T5?
*Epilobium ciliatum*	G5T5
*Geum geniculatum*	G1G2
*Geum radiatum*	G2
*Glyceria nubigena*	G2
Helianthemum bicknellii*	G5
*Houstonia purpurea* var. *montana*	G5T2
Huperzia appalachiana*	G5
Huperzia selago*	G5
*Hypericum graveolens*	G3
*Hypericum mitchellianum*	G3
*Krigia montana*	G3
*Liatris helleri*†	G2Q
*Lilium grayi*	G3
Lilium philadelphicum*	G5T4T5
Minuartia groenlandica*	G5
Packera schweinitziana*	G5?
*Phlox subulata*	G5
Platanthera grandiflora*	G5
*Platanthera peramoena*	G5
Platanthera psycodes*	G5
Poa palustris*	G5
Polygonum cilinode*	G5
*Prenanthes roanensis*	G3
Rhytidium rugosum*	G5
*Rugelia nudicaulis*	G3
Sibbaldiopsis tridentata*	G5
Spiranthes ochroleuca*	G4
*Stachys clingmanii*	G2
Trisetum spicatum*	G5
*Xanthoparmelia monticola*	G2?

* Species of northern distribution; includes species with presumed post-Pleistocene relictual distribution.

† From historical collections; now extirpated from Roan Mountain.

Current taxonomy and conservation status from *NatureServe Explorer*: http://www.natureserve.org/explorer/ (accessed 16 May 2013).

Conservation status reflects the scheme of the Nature Conservancy. G = global conservation status, with numerical rankings as follows: 1 = critically imperiled; 2 = imperiled; 3 = vulnerable; 4 = apparently secure; 5 = secure. A ‘range rank’ (G#G#) indicates a range of status uncertainty. T denotes an infraspecific taxon (trinomial) ranking. An inexact numerical ranking is indicated by ‘?’. Q = questionable taxon distinctiveness.

Sources: Weigl & Knowles (1995) and White & Sutter (1999).

In addition to supporting rare plants, the balds are associated with a number of distinctive woody species and subcommunities. At the bald periphery are ecotones of invading conifers, beech (*Fagus* sp.) and other hardwoods along with groups of mountain ash (*Sorbus* sp.), green alder (*Alnus viridis*) and *Rhododendron* species; in more central areas are islands of ericaceous shrubs. Such subcommunities are now known from other temperate, montane grasslands, suggesting similar histories and conditions.

#### (f) Extant grass bald fauna

While the vertebrate and invertebrate faunas of the balds have received less attention than the flora, the balds and adjacent ecotonal communities support some rare disjuncts, migrants, and unusual combinations of southern and northern elements (Brooks, 1965). Small mammals, songbirds, salamanders, arthropods and tardigrades are especially noteworthy in this regard (Barr, 1969; Robinson, 1981; Mansfield & Roe, 1984; Nelson & McGlothlin, 1993). Balds that have grown over appear to suffer a loss of vertebrate diversity (P.D. Weigl, unpublished data).

#### (g) Current dependence on mowing and grazing management

The only balds that are currently open and relatively stable are those that have been, or are being, grazed by livestock, or that are maintained by cutting and mowing (DeSelm & Murdock, 1993; White & Sutter, 1999). All the rest are disappearing along with their dependent organisms. While one often hears grave warnings about herbivory extirpating rare flora, it is interesting to note that in most areas, 150 years of agricultural grazing has not done so in the area of this investigation (Weigl & Knowles, 1995). In fact, there is evidence from recent management that grazing has facilitated the reappearance of rare elements as well as having increased the extent of open areas available to these organisms (P.D. Weigl & T.W. Knowles, unpublished observations).

#### (h) Pleistocene megaherbivores and the ecological role of extant keystone herbivores

Although the influence of recent livestock grazing has started to receive greater attention, the impact of large herbivores from the past has largely gone unrecognized. Prior to 13000 y BP, a diverse fauna occupied the southern Appalachians (Kurtén & Anderson, 1980; Russell *et al.*, 2009). Up to 20 species of large browsers and grazers are known from this region, including mammoth (*Mammuthus primigenius*), mastodon (*Mammut americanum*), horse (*Equus* sp.), muskox (*Bootherium bombifrons, Symbos cavifrons*), bison (*Bison latifrons, B. bison*), moose (*Alces alces*), elk (*Cervus elaphus*), caribou (*Rangifer tarandus*), stag moose (*Cervalces scotti*), fugitive deer (*Sangamona fugitiva*), tapir (*Tapirus veroensis*), peccaries (*Myohylus* sp., *Platygonus* sp.), and ground sloth (*Eremotherium* sp., *Megalonyx jeffersonii*). Such animals possessed a high degree of climatic tolerance, high mobility, immense strength, and diverse dietary preferences—characteristics which would have permitted them radically to modify high-elevation habitats, especially grassland–forest mosaics. The impact of these animals would not have been limited to feeding activities alone, but would also have included physical damage from trampling, thrashing, rubbing and trail-making. Nor would their influence be limited to warm conditions; they may have been present whenever plant foods were not snow covered and paths to the peaks unblocked.

Two lines of research have lent support to the idea that megaherbivores may have been a critical factor in the maintenance of the balds.

First, starting 50 years ago, excavations at Saltville, Virginia, in an area of ancient stream deposits dated between 14000 and 15000 ^14^C y BP and in the vicinity of the mountain ridges supporting these grass balds, unearthed about half of the megaherbivores mentioned above (McDonald & Bartlett, 1983; McDonald, 1990; Schubert & Wallace, 2009). A similar mammal assemblage of comparable age was already well known from Big Bone Lick to the north in Kentucky (Kentucky Geological Survey, 2012).

Second, in the recent past, an extensive literature has accumulated describing the role of modern keystone herbivores in maintaining open habitats and facilitating the survival of other grassland organisms (Owen-Smith, 1987, 1988, 1989). Areas as diverse as African savannas (McNaughton, 1984, 1994; Owen-Smith, 1987), the New Forest and Wiltshire Downs of England (Putman, 1986), Dutch salt marshes and the Camargue of southern France (Gordon & Duncan, 1988; Levy, 2011) all owe their vegetation structure and diversity to herbivores. The Piedmont prairies of the American southeast, now reduced to remnants, are currently thought to be the product of bison as well as fire (Horan, 1995). In the past few years analyses of complex landscapes on several continents have clearly shown the importance of large herbivores in maintaining open, diverse systems under various climatic and soil conditions, both now and in the past (Joern, 2005; Anderson *et al.*, *a*^2007^; Anderson, Ritchie & McNaughton, *b*^2007^; Burns, Collins & Smith, 2009; Holdo, Holt & Fryxell, 2009; Sankaran & Anderson, 2009; Bond, 2010; Sinclair *et al.*, 2010; Rule *et al.*, 2012). These studies have also demonstrated that the grazing activities of large herbivores may actually suppress fires by creating swards that limit fuel levels favouring ignition (Bond, 2010).

Consistent with the diversity of studies of individual areas just mentioned, recent research in Europe and Asia has raised far-reaching questions about the role of large herbivores and climate in determining the nature and extent of forest during post-glacial times. Such studies have questioned the persistent assumption that northern Europe supported an unbroken climax forest until human settlement, similar to the ‘artifact hypothesis’ mentioned here. In 2000, F.W.M. Vera produced a book-length treatise to test the hypothesis that northern and central Europe (and the eastern USA) were covered by continuous, climax forest until modified by anthropogenic influences. On the basis of a re-evaluation of palynological interpretations, the autecology of certain dominant tree species, an analysis of the behaviour and impact of large herbivores and seed dispersers, and studies of existing old growth areas, he flatly rejected this hypothesis in favour of the following alternative hypothesis:

… that the natural vegetation is a mosaic of grasslands, scrub, trees and groves in which large plant-eating mammals play an essential role in the process of the regeneration of trees and have a determining effect on the succession of species of trees. (Vera, 2000, p. 378).

Thus, one can envision the critical presence of large herbivores on natural grasslands in post-glacial Europe, as we have done for the eastern USA.

Such a novel analysis has prompted much debate (e.g. Birks, 2005), but the idea of the existence of pre-cultural open vegetation and the role of large mammals has persisted. Svenning (2002) responded to Vera's work and conducted a palaeoecological study of the interglacial and pre-agricultural Holocene vegetation of Europe. He concluded that open vegetation did indeed occupy a number of types of terrain and was likely maintained by large herbivores and possibly fire. He also provided an extensive list of plants that would have occupied such open habitats. Fenton (2008) extended the idea of a ‘natural origin for open landscape’ in a study of the uplands of Scotland. He emphasized the roles of progressive soil changes and herbivore activity in maintaining open areas in the absence of human influences. Finally, S.A. Zimov argued that, rather than merely being the passive victims of climate and vegetation change during the late Pleistocene, the large populations of grazers in Siberia actually maintained the grassland steppe ecosystem itself (Zimov *et al.*, 1995; Zimov, 2005). Thus, it is clear that the role of herbivores along with climate and other influences in maintaining open vegetation has received much greater attention and acceptance as a result of both local and regional studies during the past two decades.

### (2) The loss of the megafauna

The North American megafaunal community collapsed between 14800 and 13700 calendar y BP, with most species gone by 13000 y BP (Gill *et al.*, 2009). Evidence from a few sites suggests that some species may have persisted as late as 11500 cal y BP in the southeastern USA (Russell *et al.*, 2009). On the basis of skeletal remains, place names and reports from such early travelers as Lederer, Lawson, Logan, Catesby and Bartram, it is clear that substantial populations of elk, deer and bison occupied the open lands of the southeast and very likely became the dominant grazers and browsers on mountain grasslands (Dolin, 2010). By the late 18th century elk and bison had been extirpated, but by the 1830s, domestic livestock were more abundant and might well have carried on this ancient ecological process. However, it is likely that the loss of the original megaherbivores would have led to substantial reductions in the size and number of mountain grasslands.

### (3) Criticisms of the grazing hypothesis

The above information on grass bald history provides the background for our hypothesis on bald origins and persistence. The hypothesis has stimulated considerable discussion—both positive and negative. Much of the adverse commentary seems to stem from doubts about the potential impacts of herbivores on vegetation, a phenomenon that has been firmly established for decades by ecologists working on grasslands, savannas and other open habitats (Bond, 2010; Ripple, Rooney & Beschta, 2010; Sinclair *et al.*, 2010).

Three other concerns have been expressed. One stresses the limited size and cumulative areal extent of present-day bald communities. After all, landscape-scale grazing might be expected to have left larger, more widespread montane grasslands. However, we suggest that small, disjunct, high-elevation grassland isolates are precisely the pattern to be expected from a reduction in grazing pressure through time, following reduced herbivore diversity and density. Recently Knowles *et al.* (2012) presented an herbivore-based model that describes and partially explains the disjunct, mosaic distribution of extant bald communities. Another concern, related to the above, stems from the observation that some of the supposedly light-dependent, bald plants are known to occur in high-elevation forest near the balds or on similar wooded ridges (Wiser & White, 1999). Are these plants really part of a special balds flora as we suggest, or are they actually habitat generalists? We propose an alternative hypothesis: that these plants are likely remnants of bald landscapes that have been overtaken by forest succession, and survive temporarily in well-lit microhabitats. Finally, the most common issue raised by this hypothesis centres on the limited worldwide distribution of grass balds. Are balds merely temporary and special features of Appalachian geography, or do they occur in other temperate regions, subject to the conditions we propose? Accordingly, we describe two additional candidate ecosystems for the climate–herbivore hypothesis.

## IV. ADDITIONAL CANDIDATE ECOSYSTEMS

### (1) East Carpathian poloninas

In 2005, we learned of the existence of the *polonina* grasslands of the eastern Carpathian Mountains of Poland, Ukraine, and Slovakia, and the senior author had the opportunity to visit Bieszczady National Park in southern Poland to examine the polininas there. These vast ‘grass balds’ occupy unglaciated ridge lines below natural treeline on mountains 300 m lower than the southern Appalachian peaks, but 12° latitude further north (Halada, 1999; Winnicki & Zemanek, 2003). The poloninas support several dozen mountain grassland taxa (Table 2), including alpine relicts and endemics (Winnicki & Zemanek, 2003; Hajkova *et al.*, 2011), which are shade intolerant like those of the Appalachian balds. They also support communities of green alder (*Alnus viridis*), mountain ash (*Sorbus aucuparia*), and ericaceous shrubs, and are being invaded primarily by conifer and hardwood forests. The vertebrate and arthropod faunas include many rare and relict species, and, on the basis of descriptions and the illustrations of Winnicki & Zemanek (2003), some appear to be ecological equivalents of those of the Southern Appalachian balds. While the poloninas have been the subject of ongoing research for many years, most of this work has escaped the attention of other researchers most likely because, until recently, little had been translated into English. It is only the major monograph series emanating from Bieszczady National Park in southern Poland (e.g. Zemanek & Winnicki, 1999), publications from the East Carpathian Biosphere Reserve (Halada, 1999) and the general (translated) writings of Winnicki & Zemanek (2003) that have made us and others aware of the biota, climate, geology and ecology of the polonina region. This literature, discussions with park personnel and visits to the poloninas themselves revealed striking similarities between the poloninas and the Appalachian grass balds.

**Table 2:** Significant herbaceous subalpine flora of the East Carpathian poloninas, from Bieszczady National Park, Poland

Scientific name
*Adenostyles alliariae*
*Allium victorialis*
*Arnica montana*
*Athyrium distentifolium*
*Bupleurum longifolium*
*Calamagrostis villosa*
*Campanula rotundifolia* subsp. *polymorpha*
*Campanula serrata*
*Carex ornithopoda* subsp. *elongata*
*Centaurea kotschyana*
*Centaurea mollis*
*Cicerbita alpina*
*Cirsium waldsteinii*
*Delphinium elatum*
*Dianthus barbatus* subsp. *compactus*
*Epilobium alpestre*
*Euphorbia austriaca*
*Euphrasia picta*
*Festuca airoides*
*Geranium sylvaticum* subsp. *alpestris*
*Gnaphalium norvegicum*
*Hesperis nivea*
*Hieracium aestivum*
*Hieracium prenanthoides*
*Hypochoeris uniflora*
*Lathyrus laevigatus*
*Leucanthemum waldsteinii*
*Melampyrum herbichii*
*Melampyrum saxosum*
*Poa chaixii*
*Potentilla aurea*
*Prunus padus* subsp. *borealis*
*Pseudorchis albida*
*Ranunculus platanifolius*
*Ribes petraeum*
*Rumex alpinus*
*Scorzonera rosea*
*Senecio papposus*
*Solidago virgaurea* subsp. *alpestris*
*Tanacetum corymbosum* subsp. *clusii*
*Trollius altissimus*
*Veratrum album*
*Viola dacica*

Current taxonomy from the *Encyclopedia of Life* (online): http://www.eol.org (accessed 16 May 2013). Nature Conservancy conservation rankings are not available for these species, and most have not been evaluated for IUCN threat status.

Sources: Zemanek & Winnicki (1999) and Winnicki & Zemanek (2003).

The geological record, landscape history, and botanical data support the idea that these open grasslands are an ancient part of the vegetation of these mountains and were initially the result of climatic extremes. Using the 1980 data of Ralska-Jasiewiezowa on the late Pleistocene and Holocene pollen record, Winnicki & Zemanek (2003) document the long history of grasslands and forest–grass mosaics in this region as well as later human impacts. In studies of the Bile Karpaty mountain region, a grassland area with an especially high concentration of diverse and rare, heliophilous species, Hajkova *et al.* (2011) date this landscape to prehistoric times, describe later cultural influences and allude to the possible early effects of herbivores and fire.

What, then, can be said about the potential former impacts of ancient herbivores? In this case, we actually have a special source of data on the mammals that could have occupied the poloninas in the late Pleistocene and Holocene. In addition to fossil data and descriptions from the literature (Sutcliffe, 1985; Kurtén, 2007), there exists a record produced by upper Palaeolithic hunters from many parts of Europe displayed on the walls of numerous caves (Lascaux, Chauvet, Altamira, Cosquer, etc.). Bison, mammoth, wooly rhinoceros, reindeer, moose, elk, wild cattle, horse and many other species adorn the walls of these caves. Such animals could have very effectively exploited the grasslands and grass–forest mosaics of the Carpathians and, along with fire, helped keep them open. Even with a reduction in megafaunal diversity, maybe 12–15000 years ago (Stuart, 1991), many of these species of large mammals remained to graze and browse the mountains. Livestock grazing in the Bieszczady region started about 800 years ago (Winnicki & Zemanek, 2003), and this has helped to keep the poloninas open. Now, with the cessation or restriction of grazing in certain areas, the vegetational diversity and areal extent of some poloninas have started to decline (Halada, 1999).

### (2) Oregon Coast Range balds

Zald (2009) described the decline of the ‘grass balds’ of the Oregon Coast Range. We were surprised by the many similarities of these balds to the grasslands already discussed. These balds occur on low-elevation mountains (968–1249 m), well south of the maximum extent of the Cordilleran ice sheet (Fulton, 1991), and support many endemic and disjunct species (Table 3) (Merkle, 1951; Detling, 1953; Magee & Antos, 1992; Zald, 2009) that do not appear to tolerate the currently invading conifer forest. The area once supported a diverse Pleistocene megafauna comparable to the other sites we discuss (Kurtén & Anderson, 1980; Stenger, 1991). Zald (2009, p. 526) suggests that fire and agricultural grazing have kept these balds open in the recent past, but that these grasslands have been ‘long term features on the landscape’. He also cites documentation that domestic livestock grazed the balds from the 19th through the mid-20th century. It is significant to note that the neighbouring montane meadows of the Cascade Mountains of the northwest USA, which have been the subject of detailed studies of forest encroachment by Halpern *et al.* (2010), have similar histories of grazing and fire as possible bald maintenance factors.

**Table 3:** Representative herbaceous flora of the Oregon Coast Range balds, USA

Scientific name	Conservation status
*Achillea millefolium*	G5
*Agrostis pallens*	G4G5
*Allium crenulatum*	G4
*Anemone lyallii*	G4
*Arctostaphylos uva-ursi*	G5
*Bromus carinatus*	G5
*Cardamine nuttallii*	G5
*Cardamine pattersonii*	G2
*Carex hoodii*	G5
*Carex rossii*	G5
*Cerastium arvense*	G5
*Collinsia grandiflora*	G5
*Collinsia parviflora*	G5
*Danthonia californica*	G5
*Delphinium menziesii*	G4G5TNR
*Elymus glaucus*	G5
*Erythronium elegans*	G2
*Erythronium grandiflorum*	G5
*Erythronium oregonum*	G5
*Festuca idahoensis*	G5
*Festuca rubra*	G5
*Fragaria* x *ananassa* var. *cuneifolia*	GNA
*Iris tenax*	G4G5TNR
*Lomatium martindalei*	G5
*Lupinus albicaulis*	G5
*Lupinus sellulus* var. *lobbii*	G4TNR
*Luzula campestris*	G5
*Maianthemum stellatum*	G5
*Melica spectabilis*	G5
*Moehringia macrophylla*	G5
*Phleum alpinum*	G5
*Phlox caespitosa*	G4
*Pteridium aquilinum*	G5
*Rubus pedatus*	G5
*Rumex acetosella*	GNR
*Saxifraga hitchcockiana*	G1
*Senecio integerrimus*	G5
*Senecio triangularis*	G5
*Sidalcea hirtipes*	G2
*Silene douglasii*	G4
*Viola adunca*	G5
*Viola glabella*	G5

Current taxonomy and conservation status from *NatureServe Explorer*: http://www.natureserve.org/explorer/ (accessed 16 May 2013).

Conservation status key as in Table 1; NR, not yet ranked.

Sources: Merkle (1951) and Zald (2009).

## V. CURRENT STATUS AND FURTHER TESTS OF THE CLIMATE–HERBIVORE HYPOTHESIS

We have proposed this hypothesis to explain the origin and persistence of grass balds in three temperate regions, along with the existence of their special biotas. The hypothesis is largely based on historical data, the comparative method, modern inventories and analyses, and the recognition that these grasslands persist today only where there is an adequate herbivore presence, active intervention by people in the form of cutting and mowing, or perhaps, in a few cases, frequent fires. None of the other explanations reviewed previously (White & Sutter, 1999) incorporate as many aspects of the grass bald phenomenon as that proposed here.

Our hypothesis might further be supported or falsified by several types of information. Identification of additional candidate mountain grasslands with the same climatic and grazing histories could strengthen the hypothesis. On the other hand, the location and study of temperate montane grasslands with diverse, shade-intolerant floras, which are both stable and persistent, in the absence of a long history of large herbivores, episodic fire, or human intervention would require a reassessment of our hypothesis. Experimental approaches, through controlled studies of mountain grasslands exposed to various combinations of grazers and browsers could further support our hypothesis, or result in a re-evaluation of the mechanisms responsible for bald formation and maintenance.

Additionally, new historical or palaeontological data may come to light. In the 1540s, the Spanish explorer Hernando de Soto led an expedition into the Southern Appalachians from North Carolina, and in the 1560s one of his officers, Juan Pardo, traveled over the mountains as far as Saltville, Virginia (Beck, 1997). Although we have reviewed the translated chronicles from these expeditions (Clayton, Knight & Moore, 1993), we have found no descriptions of the local vegetation. However, it is possible that there may exist undiscovered records that could shed light on the appearance of the mountain peaks at this time, and thus on the antiquity of the balds.

In addition, new discoveries of fossil pollen, fungal spores, phytoliths and charcoal in soils and sediments could reveal different processes and time sequences for bald formation and history than those we propose here.

## VI. CONCLUSIONS

1. A group of north-temperate montane grasslands, often known as balds or poloninas, which support highly diverse, light-dependent plant communities along with an array of resident and seasonal animal populations is disappearing worldwide as a result of woody plant invasion. Long-standing debate about the origins and maintenance of these grasslands—whether they are natural ecosystems or human artifacts—has confounded conservation initiatives that would preserve these landscapes and their special biotas.
2. Historical records indicate that some North American balds pre-date European settlement. Their high species richness of shade-intolerant, rare, relictual and endemic plants is best explained by a long evolutionary history of open conditions.
3. Three such ecosystems—southern Appalachian grass balds, East Carpathian poloninas, and the Oregon Coast Range balds—are candidates for a new, unified hypothesis for origins and maintenance, involving climate and the activities of mammalian herbivores. Each of these grasslands occupies peaks well south of the maximal advance of Pleistocene ice sheets, and each is well within the known range of diverse, abundant mammalian mega- and meso-herbivores.
4. Our hypothesis posits that these grasslands owe their origin to forest suppression during maximal glacial advances, which replaced forest or woodland with open grasslands and alpine tundra. During subsequent periods, mammalian grazers and browsers, perhaps along with occasional fire, suppressed woody plant invasion, maintaining the full-sun conditions needed for the evolution of shade-intolerant plant communities, and thereby preserving the balds as long-term landscape features. The grazing hypothesis fills a gap in our understanding of the long-term persistence and dynamics of a globally significant reservoir of biodiversity.
5. Our hypothesis further posits that the end-Pleistocene megaherbivore extinctions resulted in significant woody invasion of many grasslands, although remaining herbivores such as deer, elk and bison continued to keep some areas open, albeit much reduced in areal extent. Continued reduction in native herbivore diversity and density throughout the Holocene restricted these grasslands to a few scattered peaks. Subsequently, some of these grass balds were maintained by the activities of European pastoralists, whose domestic animals acted as ecological surrogates for the extirpated native grazers.
6. We emphasize that the patterns of past and current impacts of climate and herbivores on grass balds of the Appalachians appear to be paralleled by trends in the grasslands of the East Carpathian mountains and the Oregon Coast Range, where similar conditions have promoted and preserved similar biotas and communities.
7. If this climate–herbivore hypothesis is plausible, then it may be time to rethink bald conservation in terms of preserving a natural community degraded by the absence of the ancient disturbance regime of herbivory. Only restoration of the balds will protect their rare biotas, vistas and historical places. It is also likely that, because of recent changes to these landscapes and their possible resistance to returning to their original state (Suding, Gross & Houseman, 2004), preservation in some areas will probably require a combination of management techniques, including cutting, mowing and fire. However, in many landscapes, perhaps the best strategy for restoring the balds is by using herbivores in a process that makes sense both biologically and historically.
